# Numerical methodology to evaluate unipolar saturation current limit of DC corona discharge in complex geometries

**DOI:** 10.1038/s41598-022-18144-5

**Published:** 2022-08-22

**Authors:** Sangwoo Kim, Jungho Hwang

**Affiliations:** School of Mechanical Engineering, Yonsei University, 134 Sinchon-dong, Seodaemun-gu, Seoul, 03722 Republic of Korea

**Keywords:** Computational science, Plasma physics

## Abstract

The unipolar saturation current limit (${I}_{sat}$) gives an upper limit to the corona current that can be obtained from a unipolar corona discharge. Therefore, it implies a theoretical limit to the performance of unipolar corona discharge devices. However, it has not been widely used in practice because it is difficult to deal with complex discharge configurations in an analytical way. This study aims to establish and validate a numerical methodology to evaluate the maximum current, which numerically imitates the unipolar saturation current limit. It was shown that the maximum current has the same mathematical definition as the unipolar saturation current. For validation, the maximum current was compared with an analytical solution of the Poisson equation for the coaxial cylinders configuration. The differences between the maximum current and unipolar saturation current limit for the coaxial cylinders, pin-to-plane, and single wire-to-plane configurations were discussed in terms of the assumptions used in the semi-analytical derivation of the unipolar saturation current limit. The validated methodology was applied to a multiple wire-to-plane configuration, for which a semi-analytical expression of the unipolar saturation current limit has not yet been developed. The effects of geometric and operation parameters on the maximum currents of the multiple wire-to-plane configuration were analyzed. The results were regressed into a single formula.

## Introduction

A direct current (DC) corona discharge has various modes depending on the operation conditions such as an electrode configuration, magnitude and polarity of an applied voltage, and composition and velocity of a gas^1–6^. Goldman et al.^7^ categorized various types of the DC corona discharge into unipolar and bipolar corona discharges, depending on whether one or both ion polarities exist in the inter-electrode space. In a unipolar corona discharge, such as the positive Hermstein’s glow, negative Trichel pulse, and negative pulseless glow corona discharges, ionization of gas molecules occurs only in a very small region near the discharge electrode, namely the ionization region. Only charge carriers with a single polarity (i.e. unipolar) drift through the remaining inter-electrode space, namely the drift region. Charges with opposite polarity are immediately absorbed on the surface of the discharge electrode. Because the drift region is filled only with unipolar ions, the corona current results solely from the movement of the unipolar ions, and this discharge mode is called the unipolar corona discharge. As the corona current increases, however, a discharge mode transition from unipolar to bipolar corona discharge may occur under certain conditions^7,8^. In a bipolar corona discharge, such as the streamer corona discharge, a conducting plasma is produced so rapidly that charges with opposite polarity cannot be completely absorbed at the discharge electrode^7^. Consequently, plasma filaments, each of which is called a streamer, grow out of the ionization region toward the ground electrode, resulting in secondary ionization in the drift region. In other words, ions of both polarities (i.e. bipolar) coexist in and drift through the entire inter-electrode space. It should be noted that depending on the context, the terms unipolar and bipolar can refer to not only the polarity of ions as used above, but also the polarity of the applied voltage; the bipolar corona often means the simultaneous operation of the positive and negative (i.e. bipolar) corona discharges, whereas the unipolar corona refers to the operation using only one of the positive or negative (i.e. unipolar) applied voltage^9^.

The unipolar-to-bipolar discharge mode transition is of great interest in various applications of DC corona discharges because the steady, uniform, and homogeneous characteristics of the unipolar mode are utilized in many applications. The unipolar corona discharge devices usually show better performances with higher corona currents, because the amount of unipolar ions to be used increases. However, if the corona current is so high that the discharge mode shifts to the bipolar regime, the uniformity and the homogeneity of the unipolar ion distribution are lost, degrading the performance of the devices. For instance, the collection efficiency of electrostatic precipitators is significantly reduced when the discharge mode shifts from the unipolar to the bipolar regime because the charges acquired by particulate matters are neutralized^10^. Kim and Hwang^11^ showed that the discharge mode transition from the unipolar to the bipolar lowered the electric-to-kinetic energy conversion efficiency and the thrust performance of electrohydrodynamic flow generators. Recently, Wang et al.^12^ utilized corona discharges to achieve a fast electrostatic printing of multifunctional materials to be attached on the human skin. A higher applied voltage may be preferred to achieve a faster printing, whereas it is essential to maintain the discharge in the unipolar regime for homogeneous and stable printing. Therefore, it is expected that the maximum performance of the unipolar corona discharge devices is closely related to the unipolar-to-bipolar discharge mode transition, or the maximum amount of the unipolar ions that can be obtained from the discharge conditions of interest.

In this regard, Sigmond^8^ semi-analytically derived an upper limit to the corona current that can be obtained from a unipolar corona discharge, which is called the unipolar saturation current limit (${I}_{sat}$). If the experimentally measured corona current (${I}_{exp}$) exceeds ${I}_{sat}$, then the corona discharge of interest is, at least partially, bipolar. In this case, ${I}_{exp}$ does not result solely from the movement of the unipolar ions; at least a portion of ${I}_{exp}$ results from electrons, or secondary ionization in the drift region, or both of them^8^. In other words, ${I}_{sat}$ implies a theoretical limit to the performance of unipolar corona discharge devices.

To date, the evaluation of ${I}_{sat}$ has been based on the *unipolar charge drift formula*, which describes the time-dependent change in unipolar charge density along the paths of ions^8^. Using this formula, the estimation of ${I}_{sat}$ reduces to the estimation of the lengths of electric field lines distributed in the inter-electrode space^13^. Many studies have analytically calculated the shape and length of electric field lines to obtain expressions for ${I}_{sat}$ in the discharge configurations of coaxial cylinders^8^, pin-to-plane^8,14^, and single wire-to-plane^14–16^. However, it is difficult to derive analytical solutions for electric field line distributions in complex configurations. As a result, ${I}_{sat}$ has not been widely used in the field of corona discharges in spite of its potential usefulness.

Recently, Kim et al.^17^ suggested an approach to obtain ${I}_{sat}$ without using the unipolar charge drift formula, by proposing the concept of maximum current (${I}_{max}$). They described the calculation procedure to obtain ${I}_{max}$ and showed that ${I}_{max}$ had a similar value to ${I}_{sat}$ for a single wire-to-plane type corona discharge. Later, Kim and Hwang^11^ simplified the calculation procedure by omitting the step of obtaining the size of the ionization region, which requires several iterative simulations.

This study aims to establish, validate, and apply a numerical methodology to evaluate ${I}_{max}$ for a complex discharge configuration. The calculation procedure to obtain ${I}_{max}$ was adopted from the method proposed by Kim and Hwang^11^. The procedure was validated by comparing the calculated values of ${I}_{max}$ with an analytical solution of the Poisson equation for coaxial cylinders configuration. After validation, this methodology was applied to pin-to-plane and single wire-to-plane configurations, and the values of ${I}_{max}$ were compared with those of ${I}_{sat}$ given by Sigmond^8,15^. Finally, ${I}_{max}$ of a multiple wire-to-plane configuration was evaluated. The dependence of ${I}_{max}$ on various geometric parameters and applied voltage was examined for this configuration.

## Numerical methods

### Unipolar corona discharge simulation

Corona discharge is a complex multi-physical phenomenon, and various processes are underlying, including ionization of neutral molecules by collision with electron, chemical reactions between numerous charged and neutral species, and transports of those discharge-induced species by electric field, ambient flow and ionic wind. Accordingly, numerical modeling of corona discharge in principle requires transport equations for electron, electron energy, and each species, and a set of reactions describing the chemical behavior of each species^5,18^. For a unipolar corona discharge, however, a drastically simplified model can be employed. The simplified model considers only one species: air ion. The air ion is a fictitious unipolar ion which represents various charged species generated from the unipolar corona discharge. Thus, all electrical behaviors of charged species in the unipolar corona discharge are implied in the behavior of the air ion. Within the simplified model, the generation and transport of neutral species are not considered. Therefore, chemical reactions occurring between various charged and neutral species are also not solved. Instead, the effect of the chemical reactions is implicitly included in the boundary condition of unipolar ion charge density. In the same manner, the behavior of electrons is also not considered. Thus, the potential effects of high gradient of electric field on the electron drift velocity and local field approximations cannot be discussed with the simplified model.

The simplified model consists of the steady-state governing equations for the electric field and distribution of the air ion given as follows:

1 $$\nabla^{2} V = - \frac{q}{\varepsilon },$$

2 $$\nabla \cdot \vec{j} = \nabla \cdot \left( {q\mu \vec{E}} \right) = 0,$$

where $V$ is the electric potential, $q$ is the space charge density of the unipolar air ions generated by the corona discharge, $\overrightarrow{j}$ is the unipolar corona current density, $\mu$ is the electrical mobility of the ions, and $\overrightarrow{E}=-\nabla V$ is the electric field.

For the boundary condition of $V$, constant values were imposed to the surfaces of discharge ($V={V}_{a}$) and ground ($V=0$) electrodes, respectively. To specify the boundary condition of $q$ at the discharge electrode, the ionization region was assumed to be circular and concentric with the center of curvature of the discharge electrode. The radius of the ionization region (${R}_{i}$) was assumed to be constant. For a pin-type discharge electrode, the ionization region was assumed to be a sphere with radius ${R}_{i}$, concentric with the center of curvature of the tip of the pin. Similarly, for a wire-type discharge electrode, the ionization region was assumed to be a cylinder with radius ${R}_{i}$, coaxial with the central axis of the wire. Then, a uniform and constant charge density ${q}_{i}$ was specified in the ionization region. At the ground electrode, the zero Neumann condition ($\partial q\text{/}\partial n=0$) was imposed. After the $V$ and $q$ fields converged, the corona current was calculated by integrating $\overrightarrow{j}$ over the ground electrode.

### Maximum current calculation

As mentioned in “Unipolar corona discharge simulation” section, in the simplified model, chemical aspects of corona discharge such as ionization are implicitly included in the boundary value of $q$ at the discharge electrode, ${q}_{i}$. Therefore, the problem of solving the unipolar corona discharge using the simplified model is equivalent to the problem of finding an appropriate value of ${q}_{i}$; the remaining is to solve the transport equations (Eqs. (1) and (2)) fluid-mechanically using the boundary condition. Accordingly, the unipolar corona current calculated by numerical simulation with the simplified model (${I}_{num}$) for a given applied voltage ${V}_{a}$ can be expressed as a function of ${q}_{i}$ as follows:

3 $$I_{num} = f\left( {q_{i} ;V_{a} } \right).$$

If a value of ${q}_{i}^{^{\prime}}$ is appropriately determined to represent a real corona discharge, $f$ is expected to be close to the experimentally measured corona current, that is, ${I}_{num}^{^{\prime}}=f\left({q}_{i}^{^{\prime}};{V}_{a}\right)\approx {I}_{exp}$.

The unipolar saturation current ${I}_{sat}$ is an upper limit to the corona current obtainable from the unipolar corona discharge for a given ${V}_{a}$. Intuitively, ${I}_{num}$ is expected to increase with increasing ${q}_{i}$. Therefore, in the viewpoint of Eq. (3), ${I}_{sat}$ can be expressed as follows:

4 $$I_{sat} = \mathop {\lim }\limits_{{q_{i} \to \infty }} I_{num} = \mathop {\lim }\limits_{{q_{i} \to \infty }} f\left( {q_{i} ;V_{a} } \right).$$

Kim and Hwang^11^ numerically solved the simplified model (Eqs. (1) and (2)) of the unipolar corona discharge. They fixed the value of ${R}_{i}$ and calculated ${I}_{num}$ by increasing ${q}_{i}$ stepwise by $\Delta {q}_{i}$. Their results showed that when ${q}_{i}$ was sufficiently high, ${I}_{num}$ saturated at a certain value and did not increase even though ${q}_{i}$ increased further. Kim and Hwang^11^ defined the saturated value of ${I}_{num}$ as the maximum current, ${I}_{max}$. This description is exactly identical to Eq. (4). Therefore, ${I}_{max}$ can also be written as follows:

5 $$I_{max} = \mathop {\lim }\limits_{{q_{i} \to \infty }} I_{num} = \mathop {\lim }\limits_{{q_{i} \to \infty }} f\left( {q_{i} ;V_{a} } \right).$$

In other words, ${I}_{sat}$ and ${I}_{max}$ are the semi-analytical and numerical implementations of the same expression, respectively. Thus, ${I}_{sat}$ and ${I}_{max}$ are expected to be equal to each other. In this study, ${I}_{max}$ was calculated by following the calculation procedure suggested by Kim and Hwang^11^. The flowchart of the calculation procedure is given in Supplementary Fig. S1.

## Analytical validation

Sigmond^8,15^ proposed the semi-analytical expressions of ${I}_{sat}$ for the coaxial cylinders (Eq. (6)), pin-to-plane (Eq. (7)), and single wire-to-plane (Eq. (8)) configurations as follows:

6 $$I_{sat} = \frac{2\pi \mu \varepsilon }{{R_{o}^{2} }}V_{a}^{2} \; \left[ {{\text{A m}}^{{ - {1}}} } \right],$$

7 $$I_{sat} = \frac{2.08\mu \varepsilon }{L}V_{a}^{2} \;\left[ {\text{A}} \right],$$

8 $$I_{sat} = \frac{1.62\mu \varepsilon }{{D^{2} }}V_{a}^{2} \;\left[ {{\text{A m}}^{{ - {1}}} } \right].$$

Feng^19^ reported a fully-analytical solution of the Poisson equation for corona discharge for the cylinders configuration using one-dimensional symmetricity. For other geometries, fully-analytical solutions are not available. Therefore, in this study, the calculated value of ${I}_{max}$ was validated using the current–voltage ($I$-$V$) relationship of the coaxial cylinders configuration. In “Analytical current–voltage curve for coaxial cylinders configuration” section, the fully-analytical $I$-$V$ relationship of the coaxial cylinders configuration reported in Feng^19^ was introduced. In “Maximum current calculation for coaxial cylinders configurations” section, ${I}_{max}$ was calculated for the coaxial cylinders configuration. After that, the calculated values of ${I}_{max}$ were compared with ${I}_{sat}$ and fully-analytically computed corona current, which will be denoted by ${I}_{analyt}$.

### Analytical current–voltage curve for coaxial cylinders configuration

Based on the historic works of Townsend^20^ and Thomson and Thomson^21^, Feng^19^ obtained a fully-analytical solution of the Poisson equation to express the $I$-$V$ characteristics for the coaxial cylinders configuration. Within the simplified model (Eqs. (1) and (2)), the analytical $I$-$V$ relationship for the coaxial cylinders configuration is given as follows^19^:

9 $$V_{a} = \sqrt {\left( {\frac{{I_{analyt} }}{{2\pi \varepsilon_{0} \mu }}} \right)R_{o} + C_{1} } - \sqrt {\left( {\frac{{I_{analyt} }}{{2\pi \varepsilon_{0} \mu }}} \right)R_{w} + C_{1} } + \sqrt {C_{1} } \ln \left( {\frac{{R_{o} }}{{R_{w} }}} \right) - \sqrt {C_{1} } \ln \left[ {\frac{{\sqrt {\left( {\frac{{I_{analyt} }}{{2\pi \varepsilon_{0} \mu }}} \right)R_{o} + C_{1} } + \sqrt {C_{1} } }}{{\sqrt {\left( {\frac{{I_{analyt} }}{{2\pi \varepsilon_{0} \mu }}} \right)R_{w} + C_{1} } + \sqrt {C_{1} } }}} \right],$$

where ${R}_{w}$ and ${R}_{o}$ are the radii of the inner wire and outer cylinder electrodes, respectively, and ${I}_{analyt}$ is the analytical corona current per unit length along the axial direction given as follows:

10 $$I_{analyt} = 2\pi rq\mu E.$$

${C}_{1}$ is a non-negative integral constant of the Poisson equation and expressed as follows:

11 $$C_{1} = R_{w}^{2} \left[ {E_{w}^{2} - \left( {\frac{{I_{analyt} }}{{2\pi \varepsilon_{0} \mu }}} \right)} \right],$$

where ${E}_{w}$ is the electric field strength at the surface of the inner wire electrode. Several studies employed the same approach with more details on the behaviors of electrons and ions such as ionization and attachment, and presented similar expressions to Eq. (9)^22,23^.

Because all other parameters are supposed to be given, Eq. (9) calculates the value of ${V}_{a}$ required to generate the corona current ${I}_{analyt}$ if the integral constant ${C}_{1}$ is given. To calculate ${C}_{1}$, the value of ${E}_{w}$ should first be determined. The simplest and most common method to determine ${E}_{w}$ is to apply the Kaptzov hypothesis, which states that for all ${V}_{a}$ greater than the corona onset voltage, the value of ${E}_{w}$ remains constant at the onset value ${E}_{on}$ that is usually calculated by the Peek equation. The Peek equation for a cylindrical discharge electrode at the standard temperature and pressure is given as follows:

12 $$E_{on} = 3.1 \times 10^{6} \times f \times \left( {1 + \frac{0.0308}{{\sqrt {R_{w} } }}} \right),$$

where $f$ is the roughness factor of the electrode surface.

**Figure 1:**
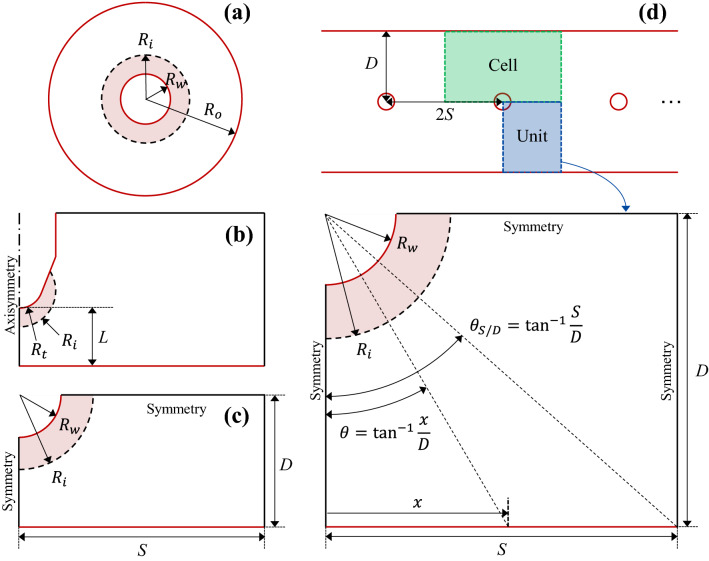
Computational domains and schematic diagrams of the ionization region: **a** coaxial cylinders, **b** pin-to-plane, **c** single wire-to-plane, and **d** multiple wire-to-plane configurations.

**Figure 2:**
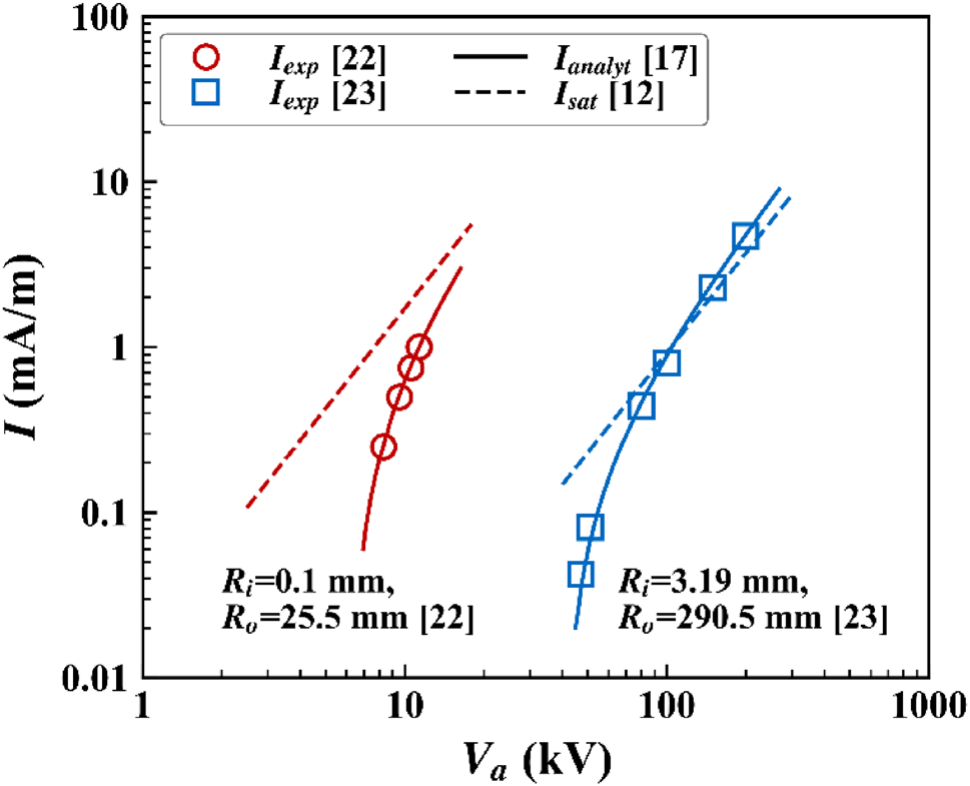
${I}_{exp}$, ${I}_{analyt}$, and ${I}_{sat}$ of the coaxial cylinders configurations: ${R}_{i}$ = 0.1 mm and ${R}_{o}$ = 25.5 mm, and ${R}_{i}$ = 3.19 mm and ${R}_{o}$ = 290.5 mm.

Two coaxial cylinders configurations were adopted for validation: ${R}_{w}$ = 0.1 mm and ${R}_{o}$ = 25.5 mm^24^, and ${R}_{w}$ = 3.19 mm and ${R}_{o}$ = 290.5 mm^25^ (see Fig. 1a). Figure 2 shows the comparisons between ${I}_{exp}$ and ${I}_{analyt}$ in the coaxial cylinders configurations. The value of $\mu$ was set to 2.0 $\times$ 10^–4^ m^2^ V^−1^ s^−1^ as reported^24,25^. The fully-analytical $I$-$V$ curve given by Feng^19^ matched with the experimental values^24,25^ with $f$ = 0.9 and 0.6, respectively. The value of $f$ is 1.0 for the polished clean surface and 0.5–0.7 for dirty scratched wires^26^. Figure 2 also shows the ${I}_{sat}$ curves for each case. For the case of ${R}_{w}$ = 0.1 mm and ${R}_{o}$ = 25.5 mm, ${I}_{exp}$ was lower than ${I}_{sat}$. It should be noted that ${I}_{exp}$ lower than ${I}_{sat}$ does not mean a unipolar corona discharge. For the case of ${R}_{w}$ = 3.19 mm and ${R}_{o}$ = 25.5 mm, ${I}_{exp}$ exceeded ${I}_{sat}$ at high ${V}_{a}$. Therefore, it can be said that for the operation condition at which ${I}_{exp}$ was higher than ${I}_{sat}$, at least a portion of ${I}_{exp}$ resulted from a bipolar corona discharge.

### Maximum current calculation for coaxial cylinders configurations

**Figure 3:**
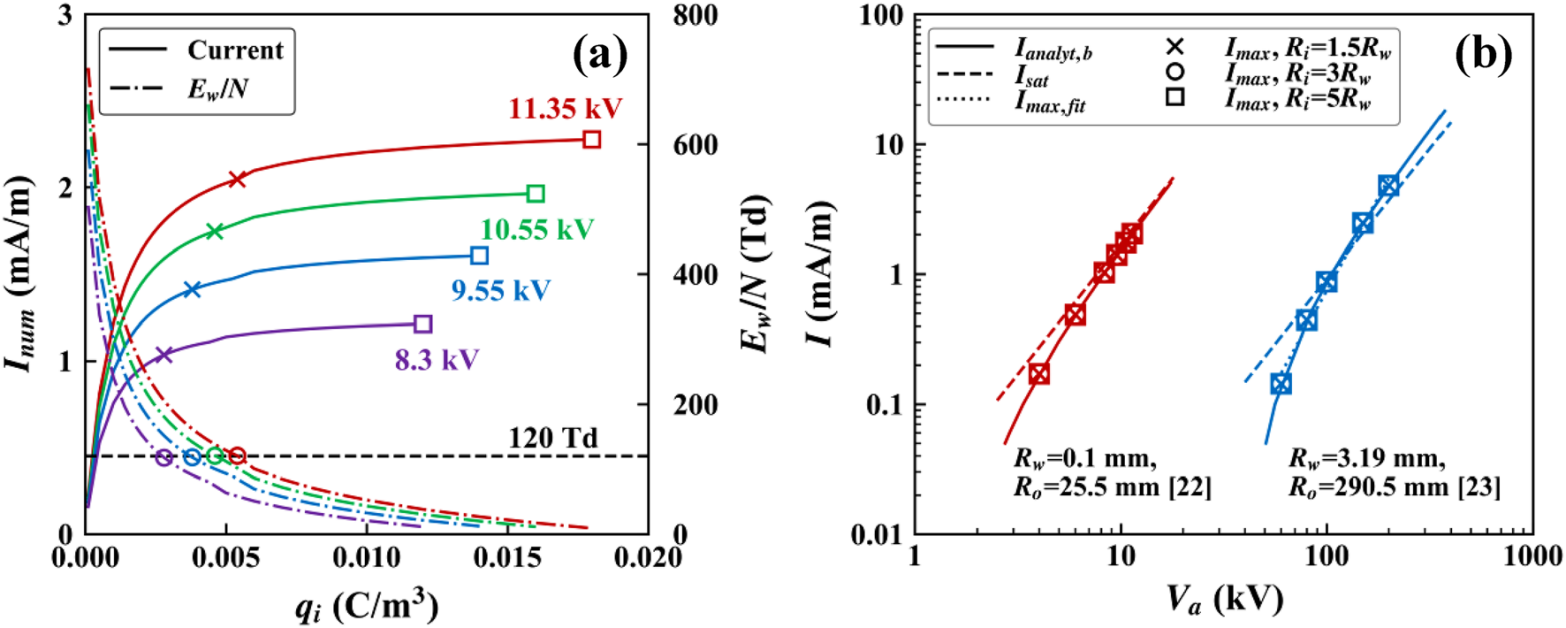
Results for the coaxial cylinders configuration: **a** saturation profiles of ${I}_{num}$ and ${E}_{w}\text{/}N$ for ${R}_{w}$ = 0.1 mm, ${R}_{o}$ = 25.5 mm, and ${R}_{i}$ = 3 ${R}_{w}$, and **b** ${I}_{anlayt,b}$, ${I}_{sat}$, and ${I}_{max}$ under various calculation conditions.

Following the procedure described in “Maximum current calculation” section, ${I}_{max}$ was calculated for the coaxial cylinders configurations. Figure 3a shows the saturation profiles of ${I}_{num}$ versus ${q}_{i}$ for positive discharge under the conditions ${R}_{w}$ = 0.1 mm, ${R}_{o}$ = 25.5 mm, and ${R}_{i}$ = 3 ${R}_{w}$. For any value of ${V}_{a}$, ${I}_{num}$ became saturated as ${q}_{i}$ increased (see square markers in Fig. 3a), as in Kim and Hwang^11^. Thus, the value of ${I}_{num}$ corresponding to the square marker can be accepted as ${I}_{max}$ for a given ${V}_{a}$. Figure 3a also shows (right vertical axis) the profiles of the reduced electric field strength at the surface of the discharge electrode (${E}_{w}\text{/}N$, where $N$ is the number density of neutral gas molecules) versus ${q}_{i}$. When ${q}_{i}$ increased, ${E}_{w}\text{/}N$ decreased while ${I}_{num}$ increased. At any saturation point, ${E}_{w}\text{/}N$ was below 120 Td (1 Td = 10^−21^ V m^2^), which is equivalent to the breakdown electric field strength (${E}_{b}$) of air at the standard temperature and pressure. The ionization coefficient is greater than the attachment coefficient where $E\text{/}N>{E}_{b}\text{/}N=120 \mathrm{Td}$ ^27,28^. Electrons are sufficiently accelerated and energetic enough to ionize the colliding neutral molecules when $E>{E}_{b}$. On the other hand, when $E<{E}_{b}$, electrons are not sufficiently accelerated. Insufficiently energetic electrons cannot ionize neutral molecules, but they are attached to the molecules. Therefore, it is reasonable to assume that ${E}_{w}$ should also remain greater than ${E}_{b}$ even if the unipolar corona discharge is in its saturated state. The circular markers in Fig. 3a indicate ${E}_{w}\text{/}N$ = 120 Td, and the cross markers indicate the corresponding ${I}_{num}$. Hereinafter, if ${E}_{w}\text{/}N$ reached 120 Td, the corresponding ${I}_{num}$ was accepted as ${I}_{max}$ even though ${I}_{num}$ was not yet completely saturated.

As mentioned in “Analytical current–voltage curve for coaxial cylinders configuration” section, the value of ${E}_{w}$ should be first specified to calculate ${I}_{analyt}$. As discussed with Fig. 2, ${E}_{on}$ was used as ${E}_{w}$ for a real corona discharge. However, for the saturated state, the representative value of ${E}_{w}$ has not been reported. In other words, it is unclear which value should be used as ${E}_{w}$ in Eq. (11) to calculate ${I}_{analyt}$ representing ${I}_{sat}$ and ${I}_{max}$. Obviously, the Kaptzov hypothesis cannot be employed for the saturated state, as shown in Fig. 2; ${I}_{analyt}$ calculated by using ${E}_{w}$ = ${E}_{on}$ was significantly different from ${I}_{sat}$ for the case ${R}_{w}$ = 0.1 mm and ${R}_{o}$ = 25.5 mm. Sigmond^8^ derived the semi-analytical expression of ${I}_{sat}$ without using information on ${E}_{w}$. Instead, an average electric field strength (e.g., $\overline{E }={V}_{a}\text{/}{R}_{o}$ for the coaxial cylinders configuration) was used. Therefore, without a better option, the breakdown electric field strength ${E}_{b}$ was used as ${E}_{w}$ for the saturated state, as also used for ${I}_{max}$. ${I}_{analyt,b}$ given in Fig. 3b indicate the value of ${I}_{analyt}$ calculated using ${E}_{w}$ = ${E}_{b}$. As shown in Fig. 3b, ${I}_{max}$ was the same as ${I}_{analyt,b}$ for all ${V}_{a}$.

The dashed lines in Fig. 3b indicate the ${I}_{sat}$ curves. ${I}_{max}$ and ${I}_{sat}$ had the similar magnitudes as in Kim et al.^17^, but with some deviations. The semi-analytical expressions for ${I}_{sat}$ given in Eqs. (6)–(8) were originally derived under various assumptions such as the Deutsch assumption, which states that the space charge does not distort the shape of the space-charge-free (i.e. Laplacian) electric field lines, but only scale the strength of the electric field; and the small discharge electrode assumption, which can be expressed as ${R}_{w}\text{/}{R}_{o}\ll 1$ ^8^. Because the Deutsch assumption is valid for the coaxial cylinders configuration, the deviation between ${I}_{sat}$ and ${I}_{max}$ might result from the invalidity of the small discharge electrode assumption. For simple comparison, ${I}_{max}$ was assumed to have the same form as ${I}_{sat}$, i.e. ${I}_{max}={C}_{2}{V}_{a}^{\gamma }$, where ${C}_{2}$ is an arbitrary constant. Then, the exponent $\gamma$ was obtained from the slope of the least-squares fit line on the logarithmic scale. For the cases ${R}_{w}\text{/}{R}_{o}=0.1/25.5=0.004$ and ${R}_{w}\text{/}{R}_{o}=3.19/290.5=0.011$, the value of $\gamma$ was 2.37 and 2.86, respectively. In other words, the smaller ${R}_{w}\text{/}{R}_{o}$ was, the closer $\gamma$ was to the semi-analytical value, 2 (Eq. (6)), which was consistent with the small discharge electrode assumption.

The effects of three different ${R}_{i}$ values (1.5 ${R}_{w}$, 3 ${R}_{w}$, and 5 ${R}_{w}$) on ${I}_{max}$ were tested. For all conditions, ${R}_{i}$ had a negligible effect on ${I}_{max}$, in agreement with Kim and Hwang^11^. For a real corona discharge, the shape and size of the ionization region are probably not constant, and the distribution of charges in the ionization region is also not uniform. Thus, numerical modeling of the real corona discharge should consider those effects. However, ${I}_{max}$ is an attempt to find a limit value of corona current obtainable under a certain applied voltage from a certain discharge configuration, not a real physical current. In the viewpoint of Eq. (5), ${I}_{max}$ is a current calculated with an infinitely high ${q}_{i}$. When the value of ${q}_{i}$ is infinitely high, the way in which the boundary condition of ${q}_{i}$ is imposed should not affect the final value of ${I}_{max}$.

## Results and discussion

The validated calculation procedure was applied to the pin-to-plane and single wire-to-plane configurations to calculate ${I}_{max}$. The results were compared with ${I}_{sat}$ curves. Then, the same methodology was applied to the multiple wire-to-plane configuration to examine the effects of various geometric parameters and applied voltage on ${I}_{max}$. The value of $\mu$ was set to 1.4 $\times$ 10^–4^ and 1.6 $\times$ 10^–4^ m^2^ V^-1^ s^-1^ for positive and negative discharges, respectively^29^.

### Pin-to-plane configuration

**Figure 4:**
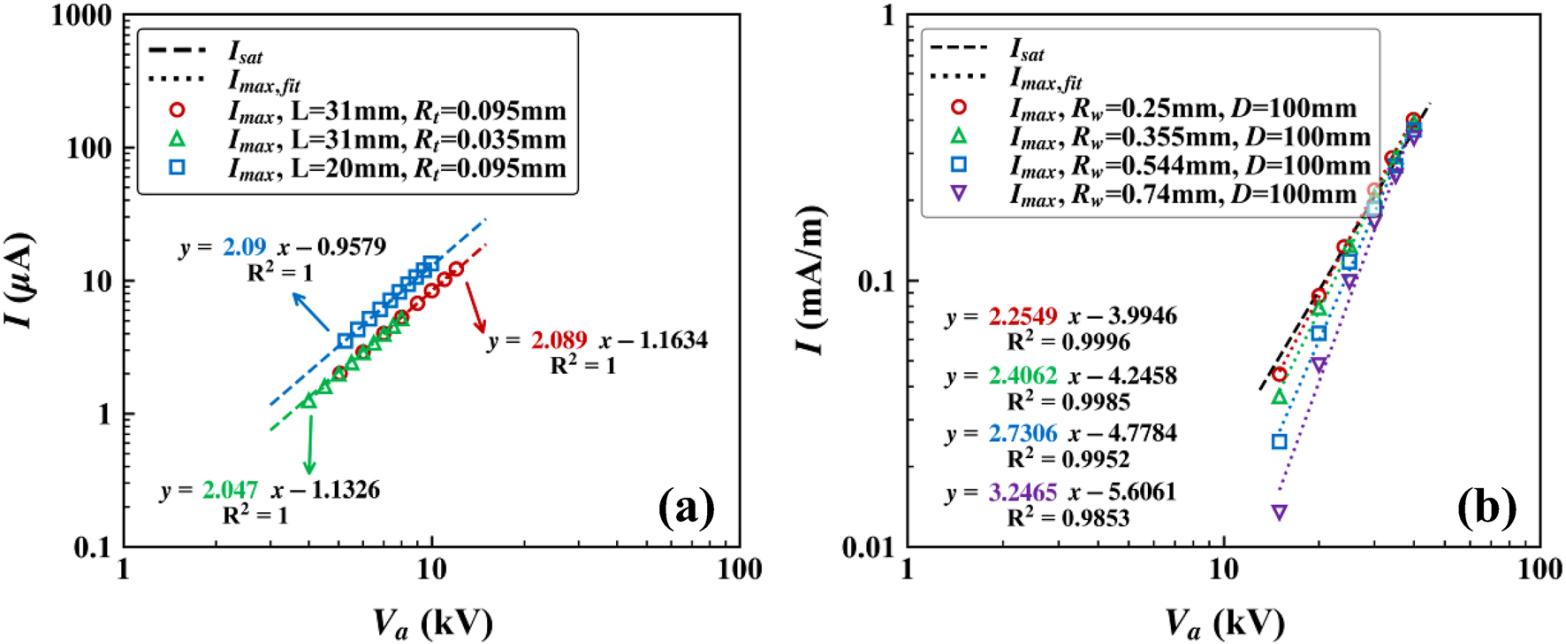
${I}_{sat}$ and ${I}_{max}$ for **a** the pin-to-plane configuration under ${R}_{i}$ = 3 ${R}_{t}$, and **b** the single wire-to-plane configurations under ${R}_{i}$ = 3 ${R}_{w}$.

The computational domain for the positive corona discharge in the pin-to-plane configuration was adopted from Adamiak and Atten^30^ (see Fig. 1b). Figure 4a shows that the values of ${I}_{max}$ were approximately the same as those of ${I}_{sat}$ in various pin-to-plane configurations. With assuming ${I}_{max}={C}_{2}{V}_{a}^{\gamma }$ as in Section Maximum current calculation for coaxial cylinders configurations, the values of $\gamma$ were given by 2.047, 2.089, and 2.090 for ${R}_{t}\text{/}L$ = 0.0011, 0.0031, and 0.0048, respectively, which were very close to the semi-analytical value, 2 (Eq. (7)). As mentioned in Section Analyticalcurrent voltage curve for coaxial cylinders configuration Based and Maximum current calculation for coaxial cylinders configurations, the semi-analytical expressions of ${I}_{sat}$ given in Eqs. (6)–(8) were derived under the small discharge electrode assumption. Therefore, it can be said that the pin electrodes with ${R}_{t}\text{/}L$ = 0.0011 to 0.0048 used in this study were small enough to neglect the effect of ${R}_{t}$ on ${I}_{max}$. It is noteworthy that unlike the coaxial cylinders configurations, the Deutsch assumption is basically not valid for the non-symmetric geometry such as the pin-to-plane and wire-to-plane configurations^31,32^. Jones and Davies^31^ concluded that the use of the Deutsch assumption to the pin-to-plane configuration should be avoided because it can cause a significant error. Nevertheless, ${I}_{max}$ was in good agreement with ${I}_{sat}$ for the considered conditions. It is unclear and has not been reported in the literature whether and how the validity of the Deutsch assumption affects ${I}_{sat}$. However, the analysis of the theoretical background of ${I}_{sat}$ is not in the scope of this study, and thus, will not be further discussed. The change in ${R}_{i}$ (1.5 ${R}_{t}$, 3 ${R}_{t}$, and 5 ${R}_{t}$) had little effect on ${I}_{max}$ of the pin-to-plane configuration as shown in Supplementary Fig. S2.

### Single wire-to-plane configuration

The computational domain for the negative corona discharge in the single wire-to-plane configuration was adopted from Ohkubo et al.^33^ (see Fig. 1c). Figure 4b shows ${I}_{max}$ versus ${V}_{a}$ under $D$ = 100 mm and various ${R}_{w}$. The values of $\gamma$ were greater than 2 for all ${R}_{w}$ and increased to greater than 3 as ${R}_{w}$ increased. Because corona discharge would be less intensive for thicker wires, ${I}_{max}$ is also expected to be smaller for thicker wires. In addition, this result was consistent with the small discharge electrode assumption; the smaller ${R}_{w}$ was, the closer ${I}_{max}$ was to ${I}_{sat}$. As in other configurations, ${R}_{i}$ had little effect on ${I}_{max}$ of the single wire-to-plane configuration (see Supplementary Fig. S3). It is noted that both ${I}_{sat}$ given in Eq. (8) and ${I}_{max}$ discussed within the single wire-to-plane configuration are the current values for one plane.

### Multiple wire-to-plane configuration

Figure 1d shows the multiple wire-to-plane configuration. $D$ and $S$ are the wire-to-ground distance and half of the wire-to-wire spacing, respectively. The normalized position along the ground electrode is represented by $x\text{/}S$, and $\theta$ is the angle of incidence. To observe the effects of various combinations of $S$ and $D$, the following parameter was used:

13 $$\theta_{{S{/}D}} = \tan^{ - 1} \left( {S{/}D} \right).$$

As mentioned in Section Single wire to plane configuration, ${I}_{sat}=1.62\mu \varepsilon {V}_{a}^{2}\text{/}{D}^{2}$ for the single wire-to-plane configuration given in Eq. (8) describes the saturation current per one plane. For the multiple wire-to-plane configuration, this is equivalent to one cell (green shading in Fig. 1d). Assuming that ${I}_{max}$ in the multiple wire-to-plane configuration exhibits similar behavior to that of the single wire-to-plane configuration, the expression of ${I}_{max}$ for one cell of the multiple wire-to-plane configuration will have the similar form with that for the single wire-to-plane, that is, ${I}_{max,cell}={C}_{3}\mu \varepsilon {V}_{a}^{\gamma }\text{/}{D}^{\beta }$, where ${C}_{3}$, $\gamma$, and $\beta$ are constants. Then, ${I}_{max}$ of the entire multiple wire-to-plane configuration can be expressed as ${I}_{max,multiple}=2{n}_{w}{I}_{max,cell}=2{n}_{w}{C}_{3}\mu \varepsilon {V}_{a}^{\gamma }\text{/}{D}^{\beta }$, where ${n}_{w}$ is the number of wires. In addition, the wire-to-wire spacing $S$ would affect the magnitude of ${I}_{max}$ by an electrical shielding. Therefore, ${I}_{max,multiple}$ will have the following form:

14 $$I_{max,multiple} = 2n_{w} I_{max,cell} = 2n_{w} \frac{{C_{3} \mu \varepsilon }}{{D^{\beta } }}V_{a}^{\gamma } g\left( S \right)\;[{\text{A m}}^{ - 1} ],$$

where $g\left(S\right)$ is a correlation function that describes the dependence of ${I}_{max}$ on $S$. The actual simulation was conducted over half of one cell, or a unit (blue shading in Fig. 1d), using a symmetry boundary condition. ${I}_{max}$ for one unit of the multiple wire-to-plane configuration (${I}_{max,unit}$) was obtained by integrating the current density over $S$ (see Fig. 1d). Then, ${I}_{max}$ for one cell was calculated by ${I}_{max,cell}=2{I}_{max,unit}$. Hereinafter, ${I}_{max}$ for the multiple wire-to-plane configuration refers to ${I}_{max,cell}$ to directly compare the maximum current with the saturation current. Thus, ${I}_{max}$ for the multiple wire-to-plane configuration is expressed as follows:

15 $$I_{max} = \frac{{C_{4} \mu \varepsilon }}{{D^{\beta } }}V_{a}^{\gamma } g\left( S \right),$$

where ${C}_{4}$ is a constant. The ranges of $S$, $D$, and ${V}_{a}$ considered were 50–1000 mm, 50–250 mm, and 45–70 kV, respectively. For simplicity, the dependence of ${I}_{max}$ on ${R}_{w}$ was not considered, and the value of ${R}_{w}$ was fixed at 1.59 mm.

**Figure 5:**
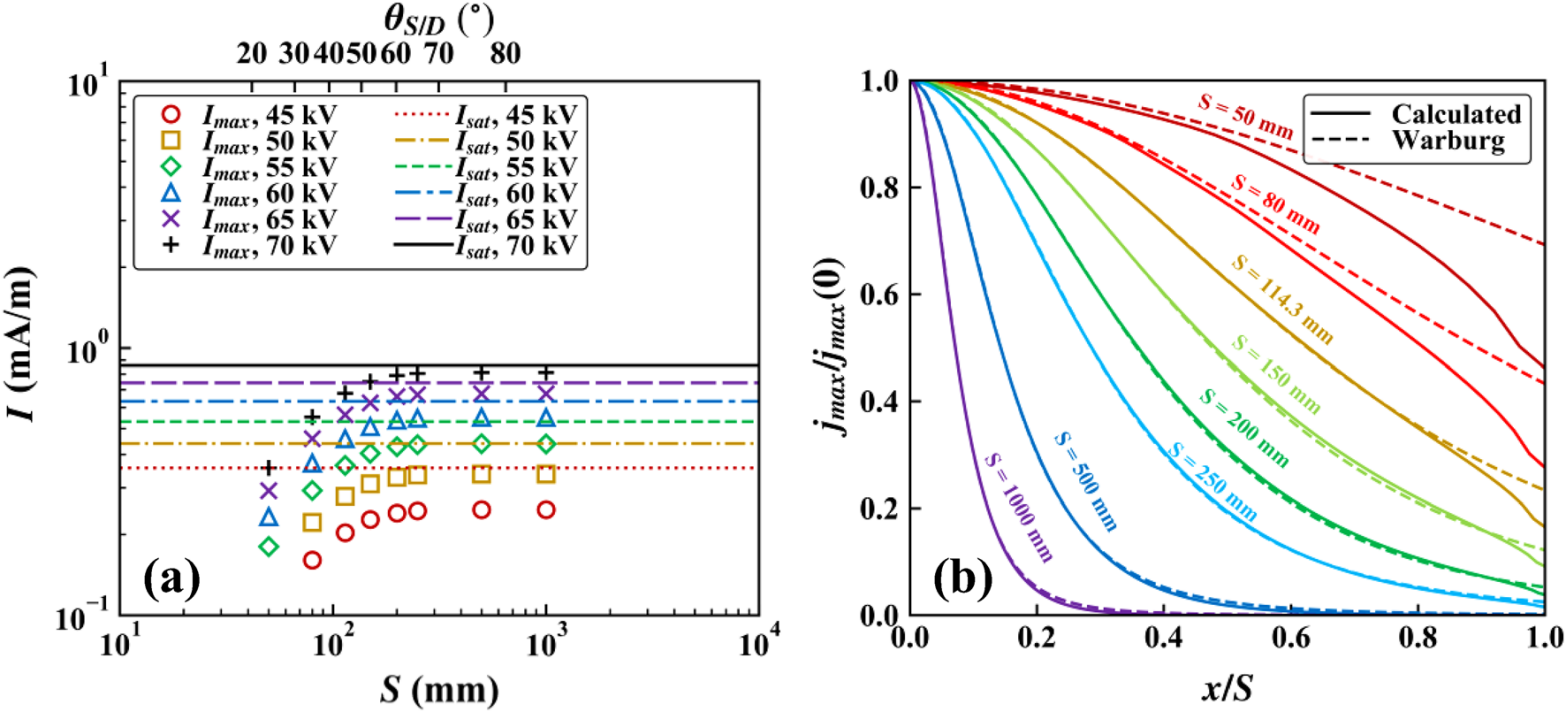
Simulation results of the multiple wire-to-plane configuration: **a** ${I}_{max}$ for various $S$ and ${I}_{sat}$ for the single wire-to-plane configuration under $D$ = 114.3 mm and various ${V}_{a}$, and **b** normalized current density distributions for ${V}_{a}$ = 60 kV and $D$ = 114.3 mm under various $S$.

Figure 5a shows ${I}_{max}$ calculated for various values of $S$ and ${V}_{a}$ with $D$ = 114.3 mm. When $S$ was small so that ${\theta }_{S\text{/}D}$ was less than approximately 65°, ${I}_{max}$ increased with increasing $S$. However, as $S$ increased further so that ${\theta }_{S\text{/}D}$ became greater than 65°, ${I}_{max}$ converged to ${I}_{sat}$ of the single wire-to-plane configuration. When ${\theta }_{S\text{/}D}$ > 65°, two adjacent wires were too far apart to affect each other. They then behaved as two independent single wire-to-plane configurations. For $D$ = 114.3 mm, $S$ = 250, 500, and 1000 mm gave ${\theta }_{S\text{/}D}$ = 65.4, 77.1, and 83.5°, respectively. In other words, for $D$ = 114.3 mm, the multiple wire-to-plane configurations with $S$ = 250, 500, and 1000 mm were equivalent to simple series of ${n}_{w}$ single wire-to-plane configurations. Figure 5a also shows ${I}_{sat}$ of the single wire-to-plane configuration for $D$ = 114.3 mm. For ${V}_{a}$ = 70 kV, ${I}_{max}$ for the multiple wire-to-plane configuration converged closely to ${I}_{sat}$ for the single wire-to-plane configuration as $S$ increased. On the other hand, for lower ${V}_{a}$, the converged value of ${I}_{max}$ was smaller than ${I}_{sat}$. This is consistent with the results for the single wire-to-plane configuration with thick wires demonstrated in Fig. 4b.

The magnitude of the corona current density ($j$) on the ground electrode is known to follow the Warburg distribution, which is given as follows^8^:

16 $$j\left( \theta \right) = j\left( 0 \right)\cos^{m} \theta \left( {\theta < 65^\circ } \right),$$

where $\theta ={\mathrm{tan}}^{-1}\left(x\text{/}D\right)$ is the angle of incidence (see Fig. 1d), and $m$ is an empirical constant. Kim et al.^17^ stated that for the single wire-to-plane configuration, the distributions of $j$ corresponding to ${I}_{exp}$ and ${I}_{num}$, namely ${j}_{exp}$ and ${j}_{num}$, followed the Warburg distribution with $m$ = 4.2. Figure 5b shows that for $S$ larger than 250 mm, the distribution of $j$ corresponding to ${I}_{max}$ (that is, ${j}_{max}$) also followed the Warburg distribution with $m$ = 4.2. Figure 5b also shows that for $S$ larger than 250 mm, ${j}_{max}$ dropped to zero before reaching the midpoint between two wires ($x\text{/}S$ = 1). In this situation, the two wires did not influence each other, and each cell in the multiple wire-to-plane configuration converged to the single wire-to-plane configuration, as shown in Fig. 5a. For the single wire-to-plane configuration, ions generated from a discharge electrode do not drift outside $\theta$ > 65°; hence, $j$ drops to zero at approximately $\theta$ = 65°^8,32^. For $S$ less than 250 mm, however, ${j}_{max}$ did not fall to zero before reaching $x\text{/}S$ = 1 because of the electrical shielding by the neighboring wires^34^. Therefore, in this range, ${I}_{max}$ was smaller by the undrawn area of the ${j}_{max}$ distribution, resulting in a decrease in ${I}_{max}$ with decreasing $S$, as shown in Fig. 5a. In addition, for $S$ less than 250 mm, ${j}_{max}$ itself deviated from the Warburg distribution. When $S$ = 50 mm, ${j}_{max}$ at $x\text{/}S$ = 1 was approximately 33% smaller than its value in the Warburg distribution. This further reduced the value of ${I}_{max}$.

**Figure 6:**
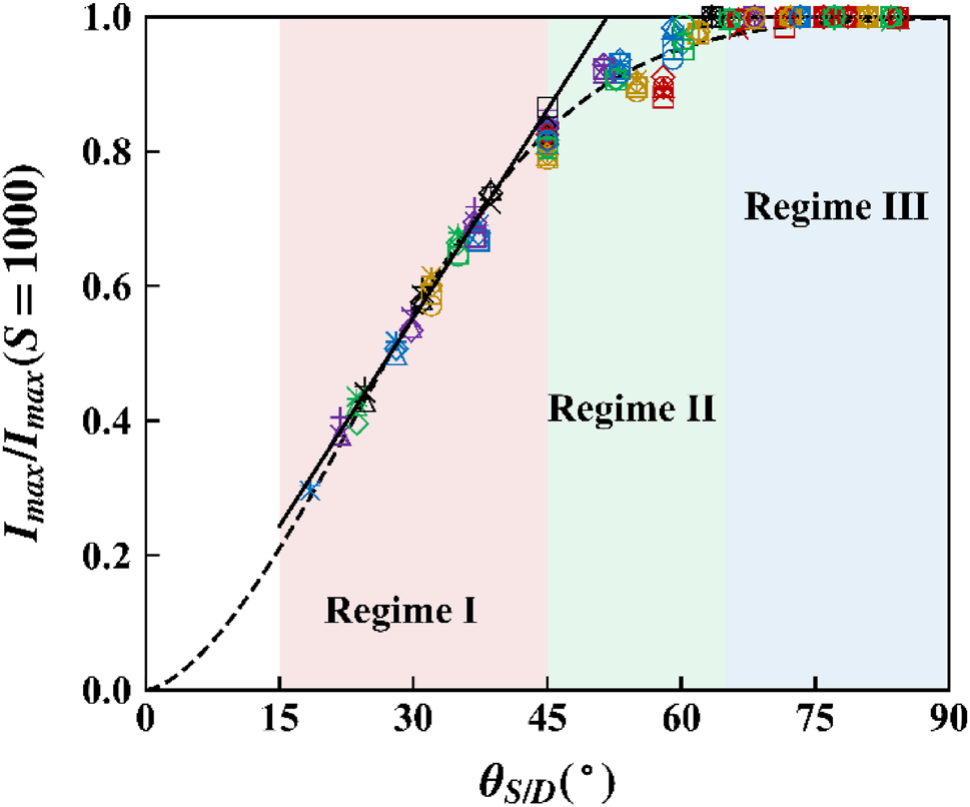
Normalized ${I}_{max}$ for various ${\theta }_{S\text{/}D}$ and ${V}_{a}$. Regime I: multiple wire, II: transition, and III: single-wire-behaved (red: $D$ = 50 mm, yellow: 80, green: 114.3, blue: 150, purple: 200, black: 250; ○: ${V}_{a}$ = 45 kV, □: 50, ◇: 55, △: 60, $\times$: 65, + : 70).

Figure 6 shows the values of ${I}_{max}$ normalized by the value of ${I}_{max}$ at $S$ = 1000 mm for various ${V}_{a}$. Note that the values of ${I}_{max}$ calculated for $S$ = 1000 mm approximate the values of ${I}_{max}$ of the single wire-to-plane configuration for the considered range of $D$ (50–250 mm). Figure 5a showed that the magnitude of ${I}_{max}$ increased with increasing ${V}_{a}$. However, Fig. 6 revealed that the normalized magnitude of ${I}_{max}$ did not depend on ${V}_{a}$; ${I}_{max}$ for different ${V}_{a}$ collapsed into a single profile. For ${\theta }_{S\text{/}D}$ < 45°, the normalized ${I}_{max}$ was linearly proportional to ${\theta }_{S\text{/}D}$. Regression analysis in the linear region (Regime I) yielded ${I}_{max}\text{/}{I}_{max}\left(S=1000\right)=1.18{\theta }_{S\text{/}D}-0.065$, where ${\theta }_{S\text{/}D}$ is in radians (solid line in Fig. 6). For ${\theta }_{S\text{/}D}$ > 65° (Regime III), ${I}_{max}$ was the same as that in the single wire-to-plane configuration, as also shown in Figs. 5a and b. Then, replacing ${I}_{max}\left(S=1000\right)$ using Eq. (15), the expression for ${I}_{max}$ is given by

17 $$I_{max} = \left\{ {\begin{array}{*{20}l} {C_{4} \left( {1.18\theta_{{S{/}D}} - 0.065} \right)\frac{\mu \varepsilon }{{D^{\beta } }}V_{a}^{\gamma } } & {{\text{for}} \quad \theta_{{S{/}D}} < 45^\circ } \\ {C_{4} \frac{\mu \varepsilon }{{D^{\beta } }}V_{a}^{\gamma } } & {{\text{for}} \quad \theta_{{S{/}D}} > 65^\circ } \\ \end{array} } \right.,$$

where ${\theta }_{S\text{/}D}$ is in radians in the regression equation. Regime II (45° < ${\theta }_{S\text{/}D}$  < 65°) represents a transition between Regimes I and III. Using regression, the three regimes can also be represented in a continuous manner as follows (dashed curve in Fig. 6):

18 $$I_{max} = C_{4} \tanh \left( {1.73\theta_{{S{/}D}}^{1.56} } \right)\frac{\mu \varepsilon }{{D^{\beta } }}V_{a}^{\gamma } ,$$

where ${\theta }_{S\text{/}D}$ is in radians.

**Figure 7:**
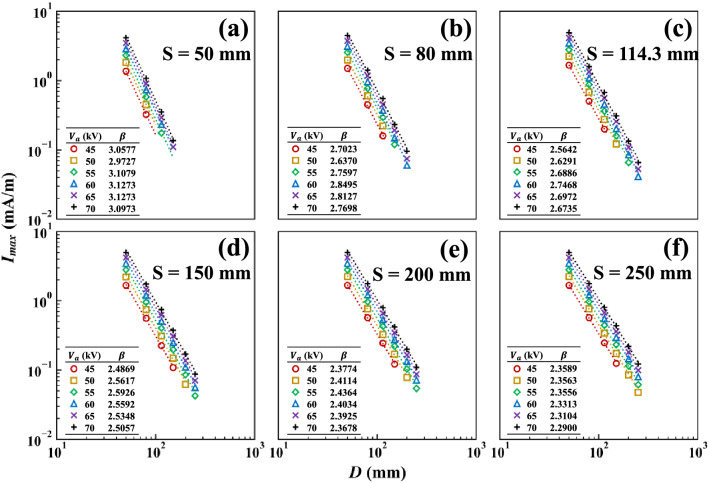
${I}_{max}$ for various $D$, $S$, and ${V}_{a}$.

Figure 7 shows the dependence of ${I}_{max}$ on $D$ for various values of $S$ and ${V}_{a}$. Taking the logarithm with respect to $D$ of both sides of Eq. (15) gave the slope of the least-squares fit line as $\beta$. For a given $S$, $\beta$ was approximately independent of ${V}_{a}$. The average values of $\beta$ were 3.06, 2.76, 2.66, 2.54, 2.40, 2.34, and 2.24 for $S$ = 50, 80, 114.3, 150, 200, 250, and 500 mm, respectively. That is, the dependence of ${I}_{max}$ on $D$ was affected by the value of $S$; the value of $\beta$ decreased and converged to that of the single wire-to-plane configuration as $S$ increased.

**Figure 8:**
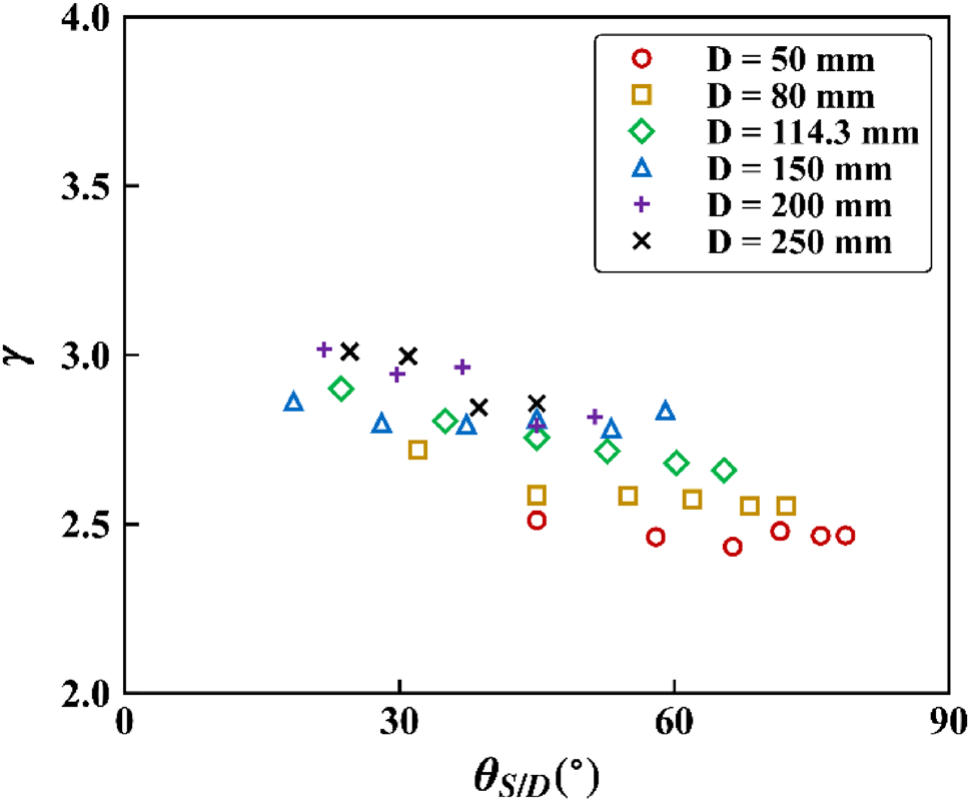
$\gamma$ for various ${\theta }_{S\text{/}D}$ and $D$.

Taking the logarithm with respect to ${V}_{a}$ of both sides of Eq. (15) gave the slope of the least-squares fit line as $\gamma$. Figure 8 shows a plot of $\gamma$ versus ${\theta }_{S\text{/}D}$ for given $D$. Note that for each value of $D$, an increase in ${\theta }_{S\text{/}D}$ corresponds to an increase in $S$. Overall, the value of $\gamma$ ranged in approximately 2.5–3.0 and decreased slightly with increasing ${\theta }_{S\text{/}D}$ or $S$.

## Conclusion

In this study, a numerical methodology to evaluate the unipolar saturation current of DC corona discharges was proposed using the concept of maximum current. It was shown that the maximum current has the same mathematical definition as the unipolar saturation current. For validation, the maximum current of the unipolar corona discharge for the coaxial cylinders configuration was compared with the analytical solution of the Poisson equation. For the coaxial cylinders and single wire-to-plane configurations, the difference between the maximum current and the unipolar saturation current decreased as the radius of the wire electrode decreased, confirming the effect of small discharge electrode assumption. For the pin-to-plane configuration, the maximum current was approximately the same as the unipolar saturation current. The calculation procedure was applied to a multiple wire-to-plane configuration, for which a semi-analytical expression of the saturation current has not yet been reported. The results showed that the maximum current was influenced by the electrical shielding from the neighboring discharge electrodes. The effects of geometric and operation parameters on the maximum currents of the multiple wire-to-plane configuration were examined, showing a potential usage for complex discharge configurations.

## Supplementary Information

Supplementary Information.

## Data Availability

The datasets used and/or analyzed during the current study available from the corresponding author on reasonable request.
