# Supplementary material for: Numerical methodology to evaluate unipolar saturation current limit of DC corona discharge in complex geometries
Source: Sci Rep. 2022 Aug 22;12:14252. doi: 10.1038/s41598-022-18144-5 (PMC9395384; doi:10.1038/s41598-022-18144-5)
Supplement: Supplementary file 1 — Supplementary Information. [file 41598_2022_18144_MOESM1_ESM.pdf]

# Numerical methodology to evaluate unipolar saturation current limit of DC corona discharge in complex geometries

## – Supplementary Materials –

Sangwoo Kim, Jungho Hwang*

School of Mechanical Engineering, Yonsei University, Seoul 03722, Republic of Korea

***Corresponding author: Jungho Hwang**

School of Mechanical Engineering, Yonsei University, 134 Sinchon-dong, Seodaemun-gu, Seoul 03722, Republic of Korea

Tel: +82-2-2123-2821, Fax: +82-2-312-2821

E-mail address: hwangjh@yonsei.ac.kr

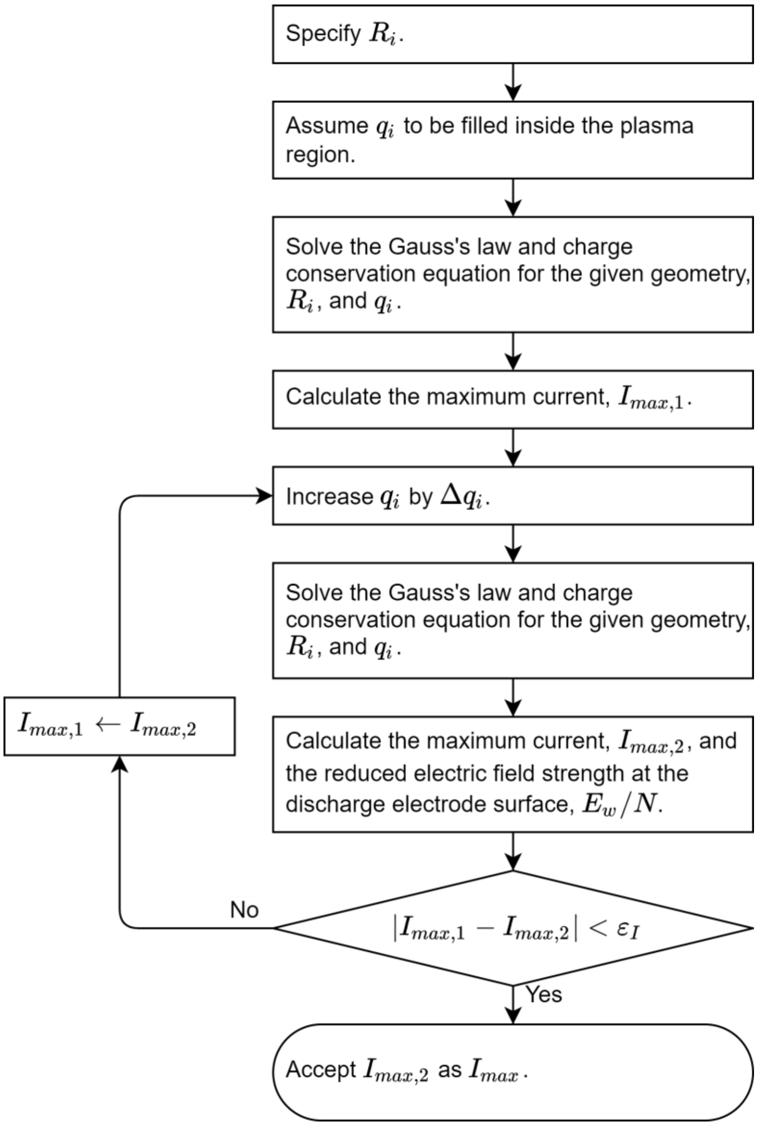


**Figure S1.** Calculation procedure to obtain the maximum current

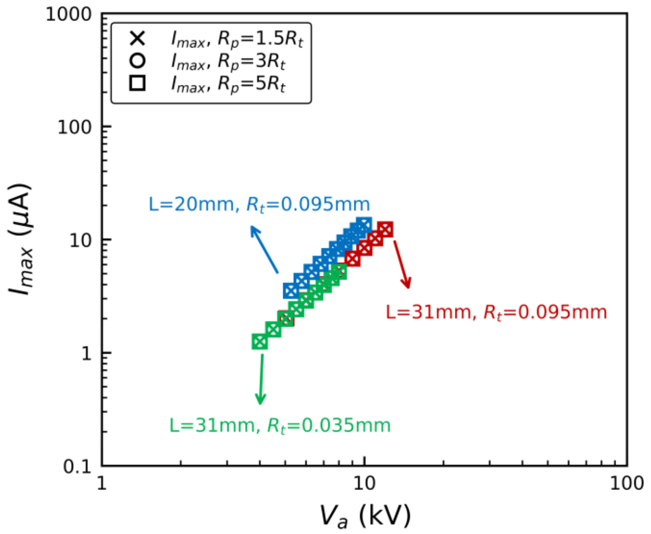


**Figure S2.** The effect of the change in $R_p$ on $I_{max}$ of the pin-to-plane configuration.

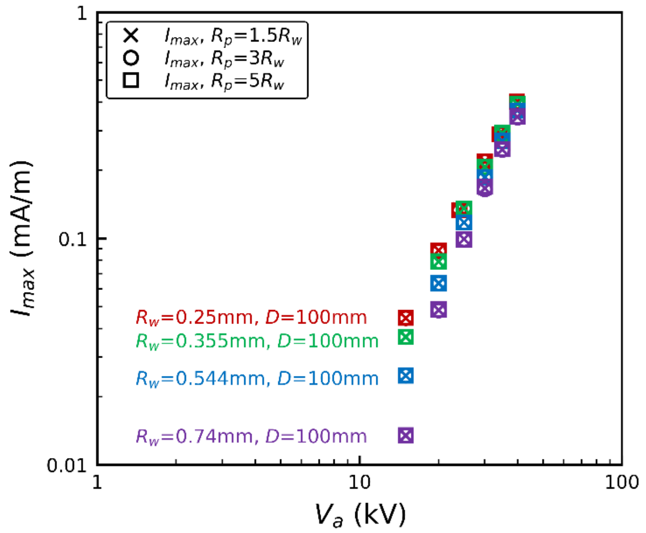


**Figure S3.** The effect of the change in $R_p$ on $I_{max}$ of the single wire-to-plane configuration.
